# Identification of therapeutic targets for inflammation in sickle cell disease (SCD) among Indian patients using gene expression data analysis

**DOI:** 10.6026/97320630014408

**Published:** 2018-07-31

**Authors:** Ipsita Das, Hrishikesh Mishra, Prafulla K. Khodiar, Pradeep K. Patra

**Affiliations:** 1 Pt. J.N.M. Medical College, Raipur, India; 2 Sickle Cell Institute Chhattisgarh, Raipur, India

**Keywords:** Sickle cell disease, vaso-occlusive crisis, inflammation, gene expression, pathophysiology, drug targets

## Abstract

Sickle cell disease (SCD) is life-threatening hemoglobinopathy prevalent in India, Sub-Saharan Africa and Middle East. Inflammation plays a pivotal role in disease process and involves intricate interaction among leukocytes, platelets, sickle erythrocytes and vascular endothelium. Available disease modifying therapies are hydroxyl-urea and blood transfusion. Therefore, it is of interest to develop improved pharmacological agents for SCD. We report up-regulated genes in steady state and vaso-occlusive crisis using analysis of gene expression data obtained by microarray experiment for SCD as potential targets. The association of these targets with inflammation in pathway analysis is also documented.

## Background

Sickle cell disease (SCD) is a life threatening hemoglobin disorder affecting about 5% of world population and is prevalent in India and other parts of the world including Sub-Saharan Africa and Middle East. In India, states namely Chhattisgarh, Odisha, Maharashtra, Madhya Pradesh and Gujarat are highly affected with this disease. In SCD, under low oxygen tension, erythrocytes become sickle-shaped due to polymerization of sickle hemoglobin (HbS) forming rigid long fibrous structures [1]. Sickle erythrocytes have difficulty in passing through the small blood vessels and block them. Target tissues become ischemic and eventually damaged. Pathophysiology of SCD involves several key factors namely hemolysis, vaso-occlusion, inflammation, and anemia [2]. Vaso-occlusion leads to acute painful episodes, which are major cause of morbidity and hospital admission. Increased inflammation has been reported as a common finding in SCD patients and is the inducer of vaso-occlusion. Finding that administration of steroids has beneficial effect during vaso-occlusive crisis (VOC) indirectly shows role of inflammatory state in pathophysiology of SCD. Inflammation plays a pivotal role in disease process and involves intricate interaction among leukocytes, platelets, sickle erythrocytes and vascular endothelium. Leukocytosis and activation of neutrophils and monocytes further increases vascular inflammation and endothelial damage and plays as a trigger for VOC [3]. In addition to vaso-occlusion, inflammation plays a pivotal role in other complications like acute chest syndrome, pulmonary hypertension, non-healing leg ulcers, nephropathy, stroke and autosplenectomy in SCD patients. Thus, inflammation culminates in a plethora of devastating acute and chronic complications causing morbidity, pain and poor quality of life among patients [4].

The only disease modifying therapies currently available are hydroxyurea [5] and blood transfusion [6]. Hydroxyurea, a ribonucleoside reductase inhibitor has been long used in pharmacotherapy of myeloproliferative disorders [7]. It has multiple mechanisms in management of SCD including induction of fetal hemoglobin (HbF), reduction in number of circulating leukocytes and reticulocytes, modulation of expression of adhesion molecules and improving rheology and flexibility of erythrocytes [8]. Hydroxyurea shown to reduce the frequency of VOC, associated pain, requirement of blood transfusions, hospitalizations and SCD related mortality. Although generally tolerated, hydroxyurea is a potentially toxic agent with low margin of safety [9]. Further, only two thirds of adult SCD patients respond to hydroxyurea therapy [10]. Blood transfusion reduces the percentage of erythrocytes having HbS but it is not universally beneficial in SCD patients. Some potential risks associated with blood transfusion are hyperviscosity, alloimmunization, autoimmunization, complement mediated hemolysis and iron overload [11]. Alloimmunization leads to delayed hemolytic transfusion reactions [12]. Furthermore, factors like availability of compatible blood and costs are also involved. Till date, hematopoietic stem cell transplantation is the only curative approach available; but it is very costly, has risks of infections, life threatening immunological reactions and is not widely used [13].

SCD has been long neglected by research community and pharmaceutical sector despite of high global burden of SCD [14]. Global burden of SCD that was 305800 in year 2010 is predicted to rise up to 404200 in year 2050 [15]. Considering the global burden of SCD, it is call of time to develop novel strategies in its management. Microarray based gene expression profiling has been long known as a powerful tool for identification of drug targets for diseases like cancers [16]. As SCD pathophysiology also involves intricate interplay of various genes, a microarray based gene expression study was done on SCD patients. This is the first microarray based gene expression study on Indian SCD patients. It is of interest to show that gene expression pattern provides insight into the patho-physiology of SCD in Indian population for mining some putative targets for intervention, suggesting effective strategies to curb the inflammatory state that is trigger for VOC and other complications; thus managing and modifying the course of disease.

## Methodology

### Sample size calculation and recruitment of subjects

Considering type 1 error rate as .05, desired fold change in expression as 2, desired power of experiment as 0.7, and standard deviation of gene intensity measurements as 0.7, sample size per group was calculated as 6. Study was done with prior approval of institutional ethics committee of Sickle Cell Institute Chhattisgarh, Raipur, India after taking informed consent from all the subjects. Recruitment of subjects was done in hospital of Sickle Cell Institute Chhattisgarh, Raipur. Twenty different subjects aged between 8-36 years, including 7 SCD patients (Hb SS) in steady state, 7 SCD patients (Hb SS) in VOC and 6 healthy controls (Hb AA) were selected for this study. Out of these subjects 4, 2 and 3 were females in abovementioned groups respectively. Subjects in steady state were defined as normally active SCD patients, not having fever and skeletal/abdominal pain at presentation and for ≥4 weeks after last VOC. Similarly subjects in VOC were defined as SCD patients having skeletal and/or abdominal pain without clinical or radiological evidence of osteomyelitis or surgical abdomen. Exclusion criteria included concomitant hydroxyurea and/or immunosuppressant therapy, renal or hepatic insufficiency, other SCD related complications, blood disorder other than SCD, pregnancy and history of blood transfusion within preceding 3 months.

### RNA isolation and microarray experiment

Five milliliters of peripheral venous blood was collected from each subject. RNA was isolated using QIAamp® RNA Blood Mini Kit (Qiagen) and its quality was assessed using Agilent 2100 Bio-analyzer, version G2938C. cDNA synthesis, cRNA synthesis and cyanine 3 (Cy3) labeling was done using Agilent one color RNA spike-In kit and Agilent Quick Amp Kit, One-Color. Quality checked Cy3 labeled cRNA samples were hybridized to microarray slides (Agilent platform GPL13497), followed by incubation of slides at 65°C for 17 hours and washing. Microarray data was captured using Agilent Feature Extraction Software (Version 9.5.3). Data pre-processing and mining were performed using GeneSpring GX (Version 12.6.1). Gene expression was compared among three subject groups using unpaired t-test and one-way ANOVA. Statistical analysis was done to find out differentially expressed genes (corrected p-value of &0.05) with minimum two-fold change in expression. Microarray based gene expression data was uploaded on Gene Expression Omnibus (http://www.ncbi.nlm.nih.gov/geo/) with accession number GSE72999.

### Validation of gene expression data through qRT-PCR

Expression levels of 4 randomly selected genes namely ICAM1, LGALS3, MAP4K5 and VCAM1 were verified through quantitative RT-PCR (qRT-PCR). Gene GAPDH was used as internal standard. cDNA was synthesized using PrimeScript first strand cDNA synthesis kit (TaKaRa Bio Inc). SYBR green chemistry (TaKaRa Bio Inc) and StepOne real time PCR system were used to quantify the transcripts. Reaction for each transcript was carried out in triplicates in a 48 well plate. Relative transcript quantities were determined using comparative CT (ΔΔCT) method [17].

### Annotation and identification of putative targets for SCD

Annotation and pathway analysis for up-regulated genes was done using GeneSpring GX (Version 12.6.1) through retrieving information from databases like WikiPathways-Analysis, Reactome, GenMAPP, BioCyc, HuGE Navigator, Entrez and GeneCards. The up-regulated genes were filtered according to their possible role in the key aspects of pathophysiology of SCD namely inflammation and activation of NF-kB pathway. NFkappa- B pathway has key implications in adhesion of leukocytes to vascular endothelium and is one of the most important regulators of pro-inflammatory genes, such as TNF alpha, IL1 beta, IL6, IL 8, IL10, interferon gamma and cyclooxygenase2. Genes were searched for instances in NCBI PubMed database also. Genes having highly generalized functions or multiple roles in various pathways were discarded. Only the genes with specific functions were selected as putative targets for intervention. Further, genes were searched for their encoded proteins, their three-dimensional structures or predicted models in protein structure/model databases.

## Discussion

### Differential gene expression

154 and 84 genes are up regulated in VOC and steady state respectively in SCD patients, compared with healthy control subjects. Pathway analyses showed that majority of genes belonged to inflammatory and stress response, cell signalling and cell cycle regulation. Similarly, 6 genes FCAR, CKAP4, MS4A4A, SLC1A3, ICA1 and ABCA1 were found to be up regulated in VOC in comparison to steady state subjects (gene expression fold change cut-off ≥2, p-value of <0.05) [18]. Except FCAR, these genes were found to be either have a wide range of actions or expressed as a response reaction to patho-physiologic milieu during VOC. Thus these genes could not qualify as putative targets. Further, gene expression pattern showed presence of aberrant inflammation, cellular stress, oxidative stress, and other cardinal features of SCD like hemolytic stress, vascular injury and repair. Among up-regulated genes, 17 and 5 genes are related to inflammation and activation of NF-kB pathway respectively.

### Validation and Cluster analysis of gene expression data

**Figure 1:**
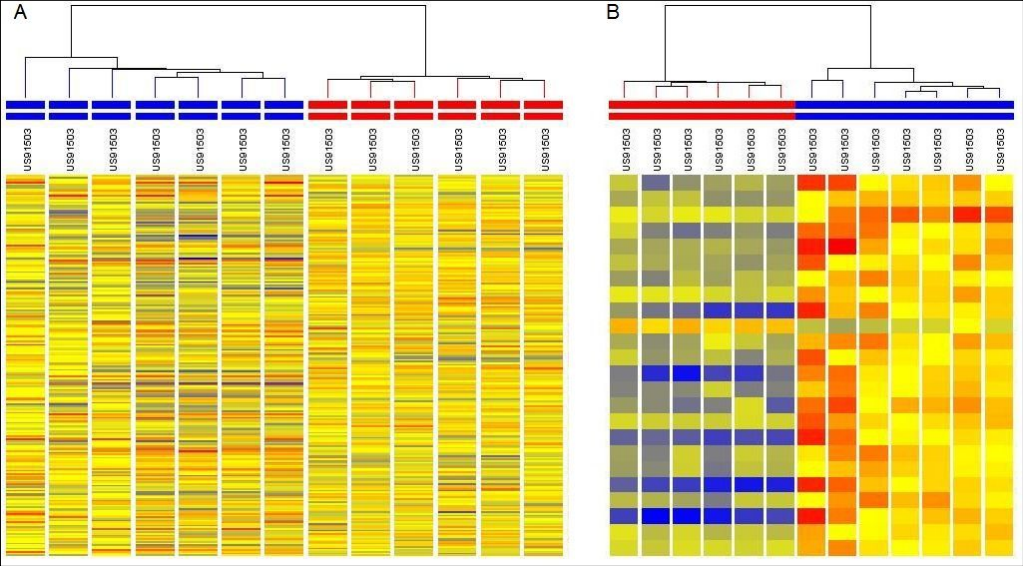
(A) Hierarchical cluster analysis of differentially expressed genes successfully separated vaso-occlusive crisis and healthy control subjects. Columns represented by red and blue coloured bars on the top represent healthy controls and vaso-occlusive crisis subjects respectively. (B) Hierarchical cluster analysis of differentially expressed genes successfully separated steady state from healthy control subjects. Columns represented by red and blue coloured bars on the top represent healthy controls and steady state subjects respectively.

Pearson correlation coefficient between gene expression fold change values in VOC & healthy control subjects and VOC & steady state subjects through microarray and qRT-PCR was found to be 0.6 and 0.9 respectively. These significant values validate the finding through microarray experiment. Further, expression data for genes differentially expressed in VOC and steady state subjects was clustered with data for healthy control subjects, using hierarchical clustering. Resultant clusters clearly separated data of six healthy controls and seven VOC subjects into two separate groups. Similarly, it also segregated data of steady state and healthy control subjects clearly. Clusters generated are shown in Figure 1.

### Mining Putative targets for intervention

Putative targets were selected as genes playing specific roles in inflammation and NF-kB pathway. Among the up-regulated genes, 5 genes playing role in inflammation and 2 genes playing role in activation of NF-kB pathway qualified as putative drug targets. Proteins encoded by these genes may be explored to mine putative targets for intervention in the disease process of SCD. These can be further confirmed by qRT-PCR and proteomic studies on SCD patients and specific interventional approaches may be designed. Three dimensional molecular structures are not available for some of these proteins, but available models and molecular modelling techniques may be used to proceed further in direction of designing drugs for inhibiting these putative targets. The details are given in Table 1.

### Putative Targets to resolve inflammation

SCD is fundamentally an inflammatory state as inflammation plays a key role in its pathogenesis [19]. It involves activation of endothelium, probably through acute effects of reperfusion injury and chronic effects by adherent erythrocytes and leukocytes [20]. Inflammation is involved in both the acute and chronic processes in SCD leading to vaso-occlusion and vascular injury. Mediators of inflammation, such as cellular adhesion molecules, cytokines, leukotrienes, and NF-kB signaling factors, represent potential therapeutic targets in SCD [21]. Five up-regulated genes known to play key role in the process of inflammation namely LGALS3, CYSLTR1, FCAR, WASF1 and VCAN, were selected as putative targets for intervention.

Galectin-3 encoded by gene LGALS3 is a lectin protein expressed by inflammatory cells. It is involved in functions like apoptosis, cell adhesion, T-cell regulation, neutrophil activation, chemotaxis of monocytes, macrophages and activation of mast cells [22]. Galectin-3 is plays pro-inflammatory role in conditions like asthma and rheumatoid arthritis and also has been considered as a potential drug target for the same [23, 24]. As SCD is considered an inflammatory condition, Galectin-3 may be a potential target for intervention in the progression of disease process in SCD too. Galectin-3 inhibitor like GMI-1051 (GlycoMimetics) may be of use in hampering the inflammation in SCD. Similarly, cysteinyl leukotriene receptor 1, encoded by gene CYSLTR1, is a G-protein coupled receptor for cysteinyl leukotrienes. Leukotrienes lead to smooth muscle contraction, increase vascular permeability, tend to sustain inflammatory reactions and may be implicated in the process of vaso-occlusion in SCD [25]. Leukotriene pathway is also implicated in bronchial asthma that is a co-morbidity associated with SCD [26]. Thus, cysteinyl leukotriene receptor 1 inhibitors like monteleukast, zafirleukast and pranlukast may be useful in reducing the inflammation and progression of disease process in SCD. Clinical trials for testing the utility of monteleukast along with hydroxyurea therapy in SCD are under process in U.S.A. Another potential strategy may be to inhibit leukotriene synthesis through use of 5-lipoxygenase inhibitor like zileuton [27]. Gene WASF1 encodes protein Wiskott-Aldrich syndrome protein family member 1, which plays a critical role downstream of Rac, a Rho-family small GTPase, in regulating the actin cytoskeleton required for membrane ruffling. Membrane ruffling is a step in leukocyte motility; so it's up regulation indicates increased inflammation [28]. WASF1 is also considered as a biomarker of traumatic as well as sub clinical brain injury that is a common finding in SCD. Thus, Wiskott-Aldrich syndrome protein family member 1 may be explored as a potential drug target in SCD, for hampering the process of inflammation. Another putative target, Versican encoded by gene VCAN is a component of the extracellular matrix and contributes in initiation of inflammatory response. Versican acts under effect of cytokines and is required in amplification of inflammatory response [29]. A study has shown that degraded C-terminal G3 fragments of versican in human plasma, also promote blood coagulation irrespective of its actions on platelets and white blood cells [30]. Versican as a component of extracellular matrix plays a central role in inflammation and as a result it is emerging as a potential target in various inflammatory conditions [31]. Therapeutic intervention to prevent or down regulate Versican may be of potential use in SCD also through halting the progression of inflammation and blood coagulation. Similarly, Immunoglobulin alpha Fc receptor, encoded using gene FCAR, acts as a receptor for Fc region of immunoglobulin alpha (IgA). The receptor is present on neutrophils, monocytes, macrophages and eosinophils and interacts with targets opsonized with IgA and triggers immunologic processes viz., release of inflammatory mediators, phagocytosis and antibody dependent cell mediated cytotoxicity [32]. It may have implications in the aberrant inflammation during VOC, and may be a potential target for intervention in the process of progression of steady state to VOC. Thus LGALS3, CYSLTR1, FCAR, WASF1 and VCAN may be potential targets to reduce inflammation in SCD.

### Putative Targets to inhibit NF-kB pathway

Monocytes in SCD patients trigger nuclear translocation of endothelial NF-kappaB (NF-kB) protein [33]. Thus, NF-kB is a critical mediator of intracellular signaling related to cellular responses to various proinflammatory signals, immune and stress response [34]. NF-kB is also an important regulator of proinflammatory genes, such as TNF alpha, IL1 beta, IL6, IL 8 and cyclooxygenase2. Inhibition of NF-kB pathway may dampen the aberrant inflammatory response in SCD and may be of use in managing the SCD patients. Some known strategies to inhibit NFkB pathway are use of sulfasalazine [35], omega-3 fatty acids [36] and zinc supplementation [37]. In our study, two genes namely UBE2V1 and TAB3, having role in activation of NF-kB pathway, were up regulated. Among these genes, UBE2V1 encodes Ubiquitin-conjugating enzyme E2 variant 1, which forms heterodimer with UBE2N and acts in concert with TRIM5 to activate the MAP3K7/TAK1 complex resulting in the increased expression of NF-kappa-B and MAPK-responsive inflammatory genes. Interleukin 1B, TNF, TRAF6 and TRAF2 also play a role in this mechanism [38]. Targeting UBE2V1 and inhibiting its heterodimerization with UBE2N may be a potential approach to inhibit activation of NF-kB pathway. Further, gene TAB3 encodes protein TGF-beta activated kinase 1, which forms a ternary complex with protein kinase MAP3K7/TAK1 and either TRAF2 or TRAF6 upon stimulation by TNF or IL-1. This process triggers a signalling cascade leading to activation of the NF-kB pathway [39]. Targeting TGF-beta activated kinase 1 and inhibiting its interaction with MAP3K7/TAK1 and either TRAF2 or TRAF6 also may be a potential approach to inhibit activation of NF-kB pathway. Thus UBE2V1 and TAB3 may be potential targets to serve as a barrier in activation of NF-kB pathway.

### Limitations of the study

Present study involved subjects available at Sickle Cell Institute Chhattisgarh, Raipur, India. Thus, all the subjects belonged to Indian population. The genes are differentially expressed in SCD patients may be different from other populations. The findings need to be checked in other populations of the world. But as about 30% of SCD patients in the world reside in India, the study may prove to be useful in its purpose. Further studies may also be done involving higher number of subjects and subjects from other parts of world affected with SCD. In present study, expression level of genes was validated through qRT-PCR study of four randomly selected genes. Only one of these genes was included in the putative targets selected through this study. Studies may be designed to validate the putative targets using qRT-PCR and proteomic techniques. Longitudinal studies may also be designed to mine differentially expressing genes in SCD patients during steady state and when they experience a VOC.

## Conclusion

Hydroxyurea is the only approved drug for SCD. It is of interest to develop novel strategies to manage SCD considering the intricate pathophysiology of this disease. Vaso-occlusion, inflammation, hemolysis, anemia, platelet hyperactivity and oxidative stress form a vicious cycle to aggravate the disease and progression. We report up-regulated genes in steady state and vaso-occlusive crisis using analysis of gene expression data obtained by microarray experiment for SCD as potential targets. These targets are found to be associated with inflammation in pathway analysis. Thus, therapeutic targets for inflammation in sickle cell disease (SCD) among Indian patients using gene expression data analysis are reported.

## Figures and Tables

**Table 1:** Putative targets for SCD with known function, structure and protein models

Gene	Protein	Function	Protein structure (in Protein Data Bank)	Predicted Protein Model (in Protein Model Portal)
LGALS3	Galectin-3	Inflammatory response, activation of mast cells	1KJL, 1KJR, 2NMO	P17931
CYSLTR1	Cysteinyl leukotriene receptor 1	Leukotriene receptor, vaso-occlusion	Not available	Q9Y271
WASF1	Wiskott-Aldrich syndrome protein family member 1	Inflammatory response	3P8C, 4N78	Q92558
VCAN	Versican	Amplification of inflammation	Not available	P13611
FCAR	Immunoglobulin alpha Fc receptor	Inflammatory response, cytokine production	1OVZ, 1OW0, 1UCT	P24071
UBE2V1	Ubiquitin-conjugating enzyme E2 variant 1	Activation of NF-Kappa B signalling pathway	2A4D, 2C2V, 2HLW	Q13404
TAB3	TGF-beta activated kinase 1/MAP3K7 binding protein 3	Activation of NF-Kappa B signalling pathway	Not available	Q8N5C8
